# Neuronal Synapse Formation Induced by Microglia and Interleukin 10

**DOI:** 10.1371/journal.pone.0081218

**Published:** 2013-11-22

**Authors:** So-Hee Lim, Eunha Park, Boram You, Youngseob Jung, A-Reum Park, Sung Goo Park, Jae-Ran Lee

**Affiliations:** Biomedical Proteomics Research Center, Korea Research Institute of Bioscience and Biotechnology, Daejeon, Korea; Institute for Interdisciplinary Neuroscience, France

## Abstract

Recently, it was found that microglia regulated synaptic remodeling of the developing brain, but their mechanisms have not been well understood. In this study, the action of microglia on neuronal synapse formation was investigated, and the primary target of microglial processes was discovered. When the developing microglia were applied to cultured hippocampal neurons without direct contact, the numbers of dendritic spines and excitatory and inhibitory synapses significantly increased. In order to find out the main factor for synaptic formation, the effects of cytokines released from microglia were examined. When recombinant proteins of cytokines were applied to neuronal culture media, interleukin 10 increased the numbers of dendritic spines in addition to excitatory and inhibitory synapses. Interestingly, without external stimuli, the amount of interleukin 10 released from the intact microglia appeared to be sufficient for the induction of synaptic formation. The neutralizing antibodies of interleukin 10 receptors attenuated the induction of the synaptic formation by microglia. The expression of interleukin 10 receptor was newly found in the hippocampal neurons of early developmental stage. When interleukin 10 receptors on the hippocampal neurons were knocked down with specific shRNA, the induction of synaptic formation by microglia and interleukin 10 disappeared. Pretreatment with lipopolysaccharide inhibited microglia from inducing synaptic formation, and interleukin 1β antagonized the induction of synaptic formation by interleukin 10. In conclusion, the developing microglia regulated synaptic functions and neuronal development through the interactions of the interleukin 10 released from the microglia with interleukin 10 receptors expressed on the hippocampal neurons.

## Introduction

Microglia are generally considered to be immunological sensors that survey neuronal diseases and viral attacks in the nerve system [1,2,3]. The roles of microglia under normal physiological conditions, however, have been relatively neglected and have not been studied as much as the pathological roles [4,5,6,7,8]. Recently, it has been reported that non-activated “resting” microglia dynamically extended and retracted their processes as if they were actively surveying the microenvironment in the brain [9,10,11]. The interaction between the fractalkine receptors (CX3CR1) in microglia and the chemokine fractalkine (CX3CL1) in neurons was suggested as having significant roles in neuronal synaptic pruning and the regulation of synaptic transmissions during postnatal development in mice [12,13]. Moreover, microglia were shown to engulf the presynaptic inputs in activity-dependent synaptic pruning processes through CR3, the receptor of the complement component C3 on microglia in the postnatal retinogeniculate system [14].

Several cytokines and growth factors released from glial cells are known to be supportive of neurons in neuronal circuit functions. Tumor necrosis factor α (TNFα), which is released from glial cells, has been considered to enhance synaptic efficacy and regulate synaptic plasticity by increasing the surface expression of glutamate receptors [15,16,17]. TNFα controlled the glutamate release step of gliotransmissions, which resulted in the induction of the presynaptic NMDA receptor-dependent synaptic potentiation [18]. ATP released from microglia also controlled the glutamate release through P2Y1 purinergic receptors on astrocytes, and increased the neuronal excitatory postsynaptic current frequency [19]. Thrombospondins, oligomeric extracellular matrix proteins released from immature astrocytes, have been identified as promoting neuronal synaptogenesis by enabling neuronal molecules to assemble into synapses during neuronal development [20]. On the other hand interleukin 1β (IL-1β) was shown to attenuate the long-term potentiation (LTP) in the hippocampus and its effects on synaptic plasticity were antagonized by interleukin 10 (IL-10) [21]. Null mutant interleukin 1 receptor (IL-1 receptor) -/- mice showed impaired learning and synaptic plasticity, but displayed a memory rescue via the transplantation of wild type neural precursor cells [22]. IL-10 has been suggested to exert neuro-protective effects and to recover neurite outgrowth by decreasing glial activation and down-regulating microglial nitric oxide (NO) production [23,24,25]. IL-10-deficient (IL-10-/-) mice were shown to be less efficient than wild type mice in a test of hippocampal-dependent learning and memory, after the intraperitoneal injection of LPS [26]. Recently, spinal cord neurons and cortical neurons were found to express interleukin 10 receptors (IL-10 receptors), and IL-10 appeared to have significant roles in neuronal development as well as neuronal protection [27,28].

As previously mentioned, resting microglia have neuronal functions, including synaptic remodeling, during the development of central nervous systems [12,13,14]. However, the primary target of these resting microglial processes has not yet been identified, and the mechanism of interaction between the microglia and the neuronal circuit is not well understood. In this paper, the induction of neuronal synapse formation by microglia was investigated using a co-culture system of hippocampal neurons and microglia without direct contact. The effects of cytokines which were known to be released from microglia were examined to find out the main factors for synaptic formation. Additionally, the expression of cytokine receptors on hippocampal neurons was discovered. To confirm the roles of cytokines, the expression of cytokine receptors was knocked down in hippocampal neurons, and then synaptic formation was analyzed. Our results suggest that microglia control neuronal synapse formation in developing neurons by releasing cytokines that interact with cytokine receptors expressed on hippocampal neurons.

## Results

### Neuronal synapse formation induced by microglia

**Figure 1:**
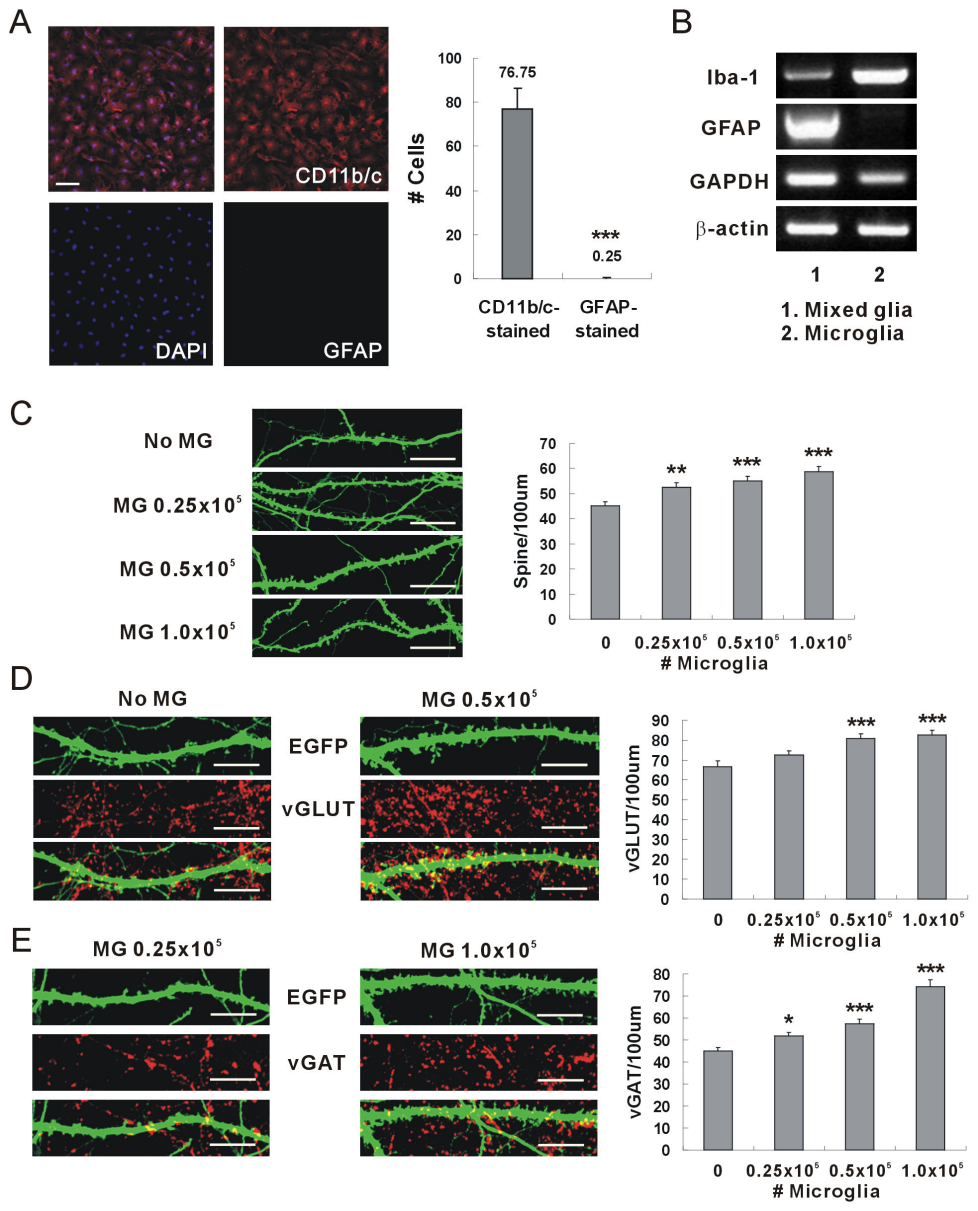
Neuronal synapse formation induced by microglia. (A) Microglia were prepared from mixed glia without astrocytes. The prepared microglia were stained with antibodies of CD11b/c (red) and GFAP (green). CD11b/c and GFAP are specific markers of microglia and astrocytes respectively. The numbers of cells stained with antibodies of CD11b/c (microglia) or GFAP (astrocytes) were counted, and the purity of microglia was analyzed. Scale bar, 10 μm. (B) An RT-PCR assay was performed to determine the purity of microglia prepared from mixed glia. The expressions of Iba-1 (a specific marker of microglia) and GFAP (a specific marker of astrocytes) were analyzed. (C) The density of dendritic spines was increased by application of microglia. Rat cultured hippocampal neurons were transfected at days in vitro (DIV) 7 with pSuper.neo-gfp, and then immunostained using anti-GFP antibodies at DIV 15 for analysis. Microglia plated on a porous cell culture insert were applied to hippocampal neurons of DIV 8. The density of dendritic spines increased when microglia was applied. Means±SEM. *n*=48 dendrites for no microglia, 48 for 0.25 × 10^5^ microglia, 48 for 0.5 × 10^5^ microglia, 48 for 1.0 × 10^5^ microglia. **p<0.01 and ***p<0.001, by the Newman-Keuls multiple comparison test after application of one-way ANOVA, *F*=9.915, *p*<0.0001. Scale bar, 10 μm. (D) The number of excitatory synapses was increased by application of microglia. The cultured hippocampal neurons were immunostained using antibodies of GFP (green) and vGLUT, the excitatory synaptic marker (red). The addition of microglia increased the number of vGLUT. Means±SEM. *n*=40 dendrites for no microglia, 40 for 0.25 × 10^5^ microglia, 40 for 0.5 × 10^5^ microglia, 40 for 1.0 × 10^5^ microglia. ***p<0.001, by the Newman-Keuls multiple comparison test after application of one-way ANOVA, F=8.206, p<0.0001. Scale bar, 10 μm. (E) The number of inhibitory synapses was increased by application of microglia. Antibodies of GFP (green) and vGAT, the inhibitory synaptic marker (red), were used for the immunostaining of hippocampal neurons. The addition of microglia increased the number of vGAT. Means±SEM. *n*=40 dendrites for no microglia, 40 for 0.25 × 10^5^ microglia, 40 for 0.5 × 10^5^ microglia, 40 for 1.0 × 10^5^ microglia. *p<0.05 and ***p<0.001, by the Newman-Keuls multiple comparison test after application of one-way ANOVA, *F*=31.81, *p*<0.0001. Scale bar, 10 μm.

Microglia were applied to hippocampal neurons to determine whether microglia could induce neuronal synapse formation without direct contact (Figure **1**). Rat pups of postnatal Day 1 were used for the preparation of developing microglia. Floating microglia were harvested, plated on porous cell culture inserts, and then applied to cultured hippocampal neurons. Previously, several components released from astrocytes were suggested to induce neuronal synapse formation [20]. Therefore, the preparation of microglia was performed very carefully to exclude astrocytes. The shape of the microglia was round and amoeboid (characteristics of “developing microglia”), when stained with anti-CD11b/c antibodies (specific marker of microglia) [29,30] (Figure **1A**). The staining with anti-GFAP antibodies (specific astrocyte marker) showed that the prepared microglia were not contaminated with astrocytes. Moreover, the prepared microglia did not express mRNA of GFAP when RT-PCR was performed, and this again confirmed their purity (Figure **1B**). 

The effect of the developing microglia on synaptic formation was examined by visualizing the dendritic spine on hippocampal neurons that were transfected and overexpressed with green fluorescent protein (GFP). The microglia plated on the porous cell culture insert were applied to the cultured hippocampal neurons of days in vitro (DIV) 8, and the synaptic formation was analyzed at DIV 15. The density of the dendritic spines was significantly increased by addition of microglia compared with the control (no microglia) (Figure **1C**). For the analysis of the excitatory and inhibitory synapses, the hippocampal neurons were stained with anti-vGLUT or anti-vGAT antibodies (Figures **1D, 1E**, respectively). The numbers of excitatory (vGLUT-stained) and inhibitory (vGAT-stained) synapses were also significantly increased by addition of microglia compared with the control.

Recently, it was found that ATP released from microglia acts on astrocytes through P2Y1 purinergic receptors, which in turn regulate synaptic activity [19]. Reactive blue (RB), the antagonist of P2Y receptors, was added to the co-culture system of microglia and hippocampal neurons to eliminate the induction of synaptic formation through P2Y receptors (Figure **S1**). Synaptic formation was not reduced by RB at all, and this result shows that synaptic formation was not induced through P2Y receptors on astrocytes. In conclusion, the developing microglia on porous cell culture inserts induced synaptic formation of hippocampal neurons without direct contact. 

### Neuronal synapse formation induced by IL-10 released from microglia

**Figure 2:**
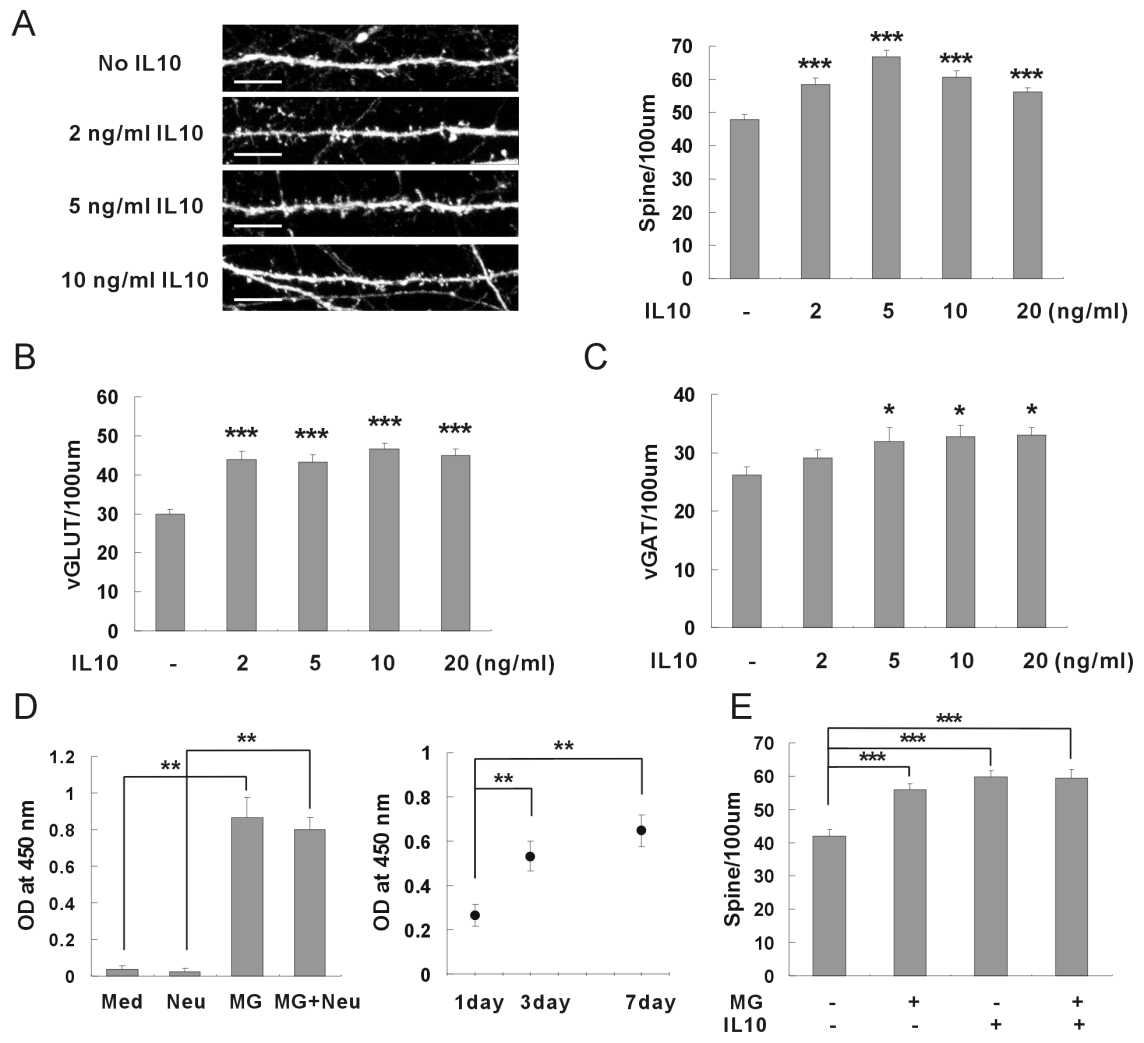
Neuronal synapse formation induced by IL-10 released from microglia. (A) The density of dendritic spines was increased by application of recombinant IL-10. Recombinant proteins of IL-10 were applied to hippocampal neurons of DIV 8 and the density of dendritic spines was analyzed after one week. Means±SEM. *n*=31 dendrites for control (no IL-10), 31 for 2 ng/ml IL-10, 31 for 5 ng/ml IL-10, 31 for 10 ng/ml IL-10, 31 for 20 ng/ml IL-10. ***p<0.001 by the Newman-Keuls multiple comparison test after application of one-way ANOVA, F=15.66, p<0.0001. Scale bar, 10 μm. (B) The number of excitatory synapses was increased by application of recombinant IL-10. The application of recombinant IL-10 increased the number of vGLUT. Means±SEM. *n*=34 dendrites for control (no IL-10), 35 for 2 ng/ml IL-10, 31 for 5 ng/ml IL-10, 31 for 10 ng/ml IL-10, 33 for 20 ng/ml IL-10. ***p<0.001 by the Newman-Keuls multiple comparison test after application of one-way ANOVA, F=16.33, *p*<0.0001. (C) The number of inhibitory synapses was increased by application of recombinant IL-10. The application of recombinant IL-10 increased the number of vGAT. Means±SEM. *n*=32 dendrites for control (no IL-10), 28 for 2 ng/ml IL-10, 29 for 5 ng/ml IL-10, 27 for 10 ng/ml IL-10, 27 for 20 ng/ml IL-10. *p<0.05 by the Newman-Keuls multiple comparison test after application of one-way ANOVA, *F*=2.960, *p*=0.0220. (D) IL-10 was released from intact microglia without external stimuli. Microglia were plated on a porous cell culture insert, and incubated in a neuronal culture media. After 3~7 days, the amount of IL-10 released from the microglia was analyzed using an ELISA kit. An appreciable amount of IL-10 was released from microglia compared with controls (neuronal culture media only or hippocampal neuron only). **p<0.01 by the Newman-Keuls multiple comparison test after application of one-way ANOVA, *F*=48.25, *p*=0.0013. (E) Microglia and recombinant IL-10 use the same signal pathway for induction of synaptic formation. Addition of recombinant IL-10 did not additively enhance the induction of synaptic formation by microglia. Means±SEM. *n*=27 dendrites for control (no microglia no IL-10), 27 for the application of 1.0 × 10^5^ microglia, 28 for 5 ng/ml of IL-10, 27 for microglia and IL-10. ***p<0.001 by the Newman-Keuls multiple comparison test after application of one-way ANOVA, *F*=15.50, *p*<0.0001.

Microglia has been known to release many kinds of cytokines in response to external stimuli [5]. Because microglia induced neuronal synapse formation without direct contact, cytokines released from microglia could be main factors for synaptic formation. Therefore, the roles of cytokines were examined by applying recombinant proteins of cytokines to hippocampal neurons (Figure **2**). When recombinant IL-10 was applied to hippocampal neurons at DIV 8 and the synaptic formation was analyzed after one week, the density of the dendritic spines was significantly increased (Figure **2A**). The numbers of excitatory synapses as well as the inhibitory synapses were also increased by application of IL-10 (Figure 2B, 2C). On the other hand, the recombinant IL-1β, TNF-α, IL-6, and IL-4 did not induce synaptic formation of hippocampal neurons at any concentrations (Figure **S2A-2D**). An ELISA assay demonstrated that an appreciable amount of IL-10 was released from the intact microglia without external stimuli in the neuronal culture media (Figure **2D**). In order to confirm the roles of IL-10 released from microglia, recombinant IL-10 was applied to hippocampal neurons together with microglia (Figure **2E**). Synaptic formation was not induced additively by application of exogenous IL-10 together with microglia. This result suggests that microglia and IL-10 use the same signal pathway for the induction of synaptic formation. In conclusion, microglia appear to induce neuronal synapse formation by releasing IL-10 into the culture media of hippocampal neurons.

### IL-10 receptors expressed on hippocampal neurons

**Figure 3:**
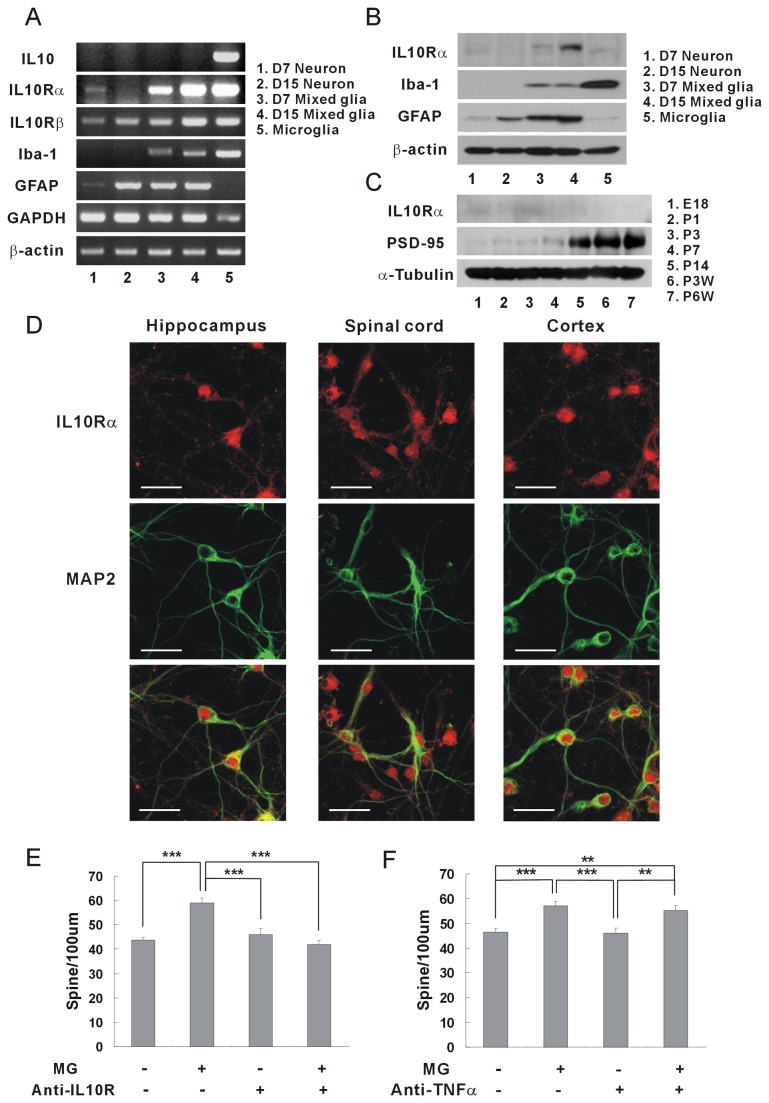
IL-10 receptors expressed on hippocampal neurons. (A) Expression of IL-10 receptor mRNAs in hippocampal neurons. The expression of the IL-10 receptor was identified using RT-PCR. The mRNAs of IL-10 receptor α and β were expressed in the hippocampal neurons. IL-10 receptor α was expressed mainly in hippocampal neurons of DIV 7. (1, cultured hippocampal neurons at DIV 7; 2, cultured hippocampal neurons at DIV 15; 3, mixed glial culture at DIV 7; 4, mixed glial culture at DIV 15; 5, microglia) Quantification (DIV 15 neuron/ DIV 7 neuron): IL-10 receptor α, 0.61; IL-10 receptor β, 1.06. (B) Expression of IL-10 receptor proteins in cultured hippocampal neurons. Similar to the expression of mRNA, the IL-10 receptor α protein was expressed mainly in neurons of DIV 7. Anti-IL-10 receptor α antibodies (0.5 μg/ml, Santa Cruz, sc-985) were used for western blotting [27]. Quantification (DIV 15 neuron/ DIV 7 neurons): IL-10 receptor α, 0.73. (C) Expression of IL-10 receptor proteins in the developing rat brains. The IL-10 receptor α proteins were expressed mainly in the developing brains of embryonic and early postnatal days (E18~P3). Quantification of IL-10 receptor α: E18, 0.30; P1, 0.27; P3, 1.0; P7, 0.22; P14, 0.20; P3W, 0.17; P6W, 0.15 (E, embryonic days; P, postnatal days). (D) Images of IL-10 receptor expressions in cultured hippocampal neurons. Hippocampal neurons of DIV 6 were stained with antibodies of IL-10 receptor α (5 μg/ml, Santa Cruz, sc-985) (red) and MAP2 (the neuronal marker, green) after treatment with 4% formaldehyde and then -20 °C methanol. Hippocampal neurons expressed IL-10 receptor proteins comparable to spinal neurons or cortical neurons. (E) The induction of synaptic formation by microglia was antagonized by the neutralizing antibody of IL-10 receptor α. When anti-mouse IL-10 receptor α antibody was applied to the co-culture of mouse microglia and mouse hippocampal neurons, the density of dendritic spines was significantly decreased compared with the control (without anti-IL-10 receptor antibody). Means±SEM. *n*=30 dendrites for no microglia without anti-IL-10 receptor antibody, 29 for the application of microglia only, 29 for no microglia with anti-IL-10 receptor antibody only, 29 for microglia with anti-IL-10 receptor antibody. ***p<0.001 by the Newman-Keuls multiple comparison test after application of one-way ANOVA, F=17.35, p<0.0001. (F) The induction of synaptic formation via microglia was not antagonized by the blocking antibody of TNFα. When anti-rat TNFα antibody was applied to the co-culture of rat microglia and rat hippocampal neurons, the density of dendritic spine was not decreased compared with control (without anti-TNFα antibody). Means±SEM. *n*=27 dendrites for no microglia without anti-TNFα antibody, 28 for the application of microglia only, 29 for no microglia with anti-TNFα receptor antibody only, 27 for microglia with anti-TNFα antibody. **p<0.01 and ***p<0.001 by the Newman-Keuls multiple comparison test after application of one-way ANOVA, *F*=9.104, *p*<0.0001.

It is well known that IL-10 receptors are expressed on glial cells [1,8]. Recently, it has been discovered that spinal neurons, cortical neurons, and retinal ganglion cells also have IL-10 receptors [27,28,31]. Using the RT-PCR assay, the expressions of IL-10 receptors were examined on cultured hippocampal neurons (Figure **3A**). Interestingly, the IL-10 receptor α was mainly expressed in early developing hippocampal neurons of DIV 7, and on the other hand, IL-10 receptor β was expressed similarly in DIV 7 and DIV 15 neurons (Figure **3A**, IL10Rα & IL10Rβ, panels 1 and 2). Because IL-10 receptors have been known to be expressed on glial cells, the question was raised whether the IL-10 receptor mRNA shown in panels 1 and 2 originated from hippocampal neurons [32]. The prepared microglia exhibited very strong expressions of IL-10 receptors, but the cultured neurons appeared not to be contaminated with microglia (Figure **3A**, no Iba-1, a specific microglia marker, panels 1 and 2). Although astrocytes appeared to grow with hippocampal neurons (Figure **3A**, GFAP, panels 1 and 2), the IL-10 receptor mRNA of the hippocampal neurons did not appear to originate from the astrocyte, as indicated by the IL-10 receptor α expression being stronger in DIV 7 neurons than in DIV 15 neurons, which is opposite to the GFAP expression (stronger in DIV 15 neurons than in DIV 7 neurons).

Then, the expressions of IL-10 receptor proteins were examined in cultured hippocampal neurons using anti-IL-10 receptor α antibody (Figure **3B**). Similar to the mRNA expressions, the IL-10 receptor α proteins were expressed mainly in DIV 7 neurons (Figure **3B**, panels 1 and 2, Mw 90-110 kDa) [33]. The developing rat brains of embryonic and early postnatal days also showed stronger expressions of IL-10 receptor α than did adult rat brains (Figure **3C**). When an immunohistofluorescence was performed using anti-IL-10 receptor antibody, the hippocampal neurons expressed IL-10 receptor α not less than spinal cord neurons or cortical neurons did (Figure **3D**).

In order to confirm the functions of IL-10 in synaptic formation, the neutralizing antibody of IL-10 receptor was applied to a co-culture system and the interaction between IL-10 and IL-10 receptor were inhibited (Figure **3E**). The induction of synaptic formation by microglia disappeared when the neutralizing antibody of IL-10 receptor was applied. However, the anti-TNFα antibodies did not inhibit the induction of the synaptic formation by microglia (Figure **3F**). As a result, synaptic formation was induced through the interaction of IL-10 released from the microglia with IL-10 receptors expressed on the hippocampal neurons of early postnatal development.

### Neuronal synapse formation attenuated by the knockdown of IL-10 receptors

**Figure 4:**
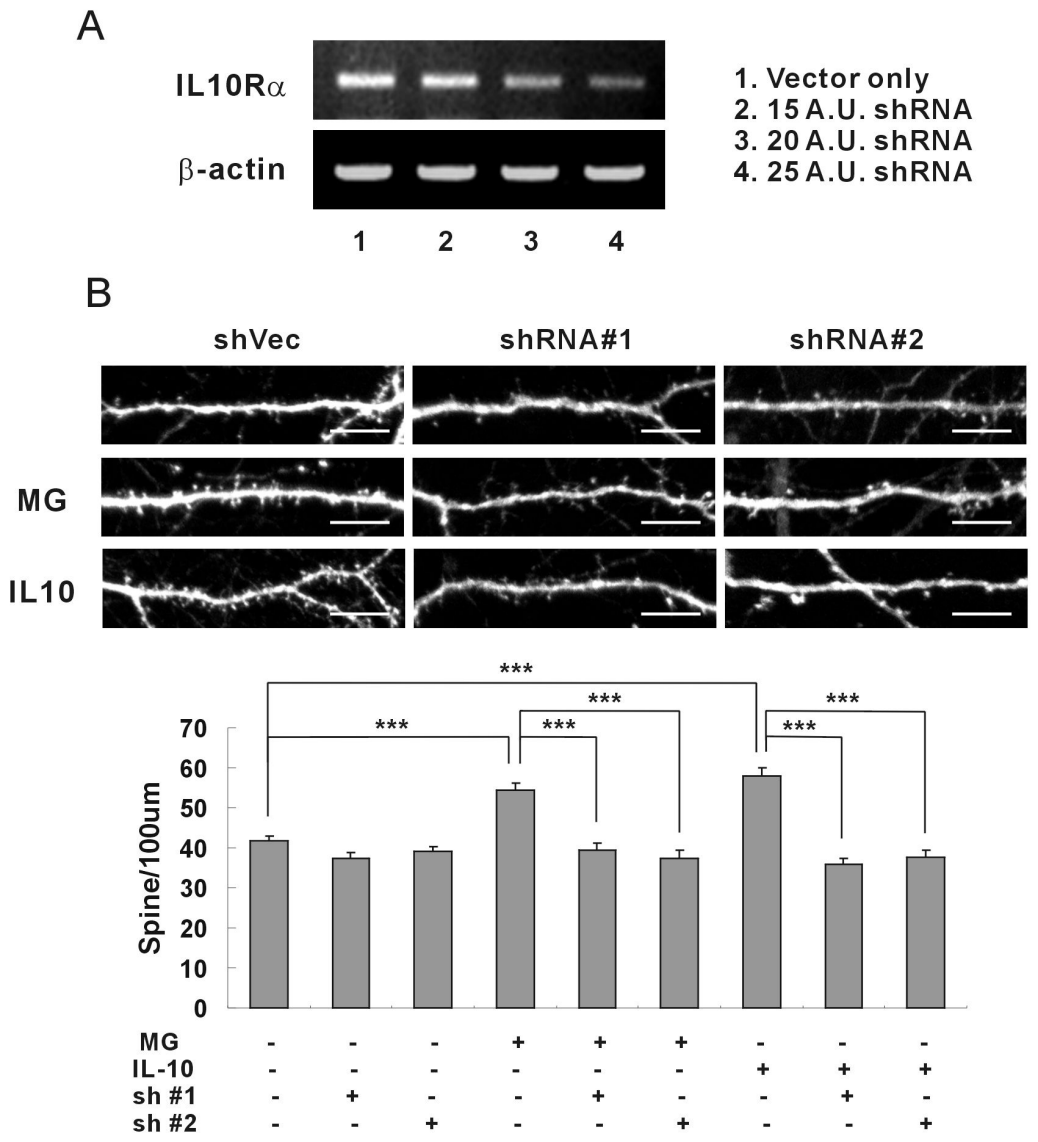
Neuronal synapse formation attenuated by the knockdown of IL-10 receptors. (A) The expressions of IL-10 receptors attenuated by shRNA in hippocampal neurons. The lentivirus expressing shRNA of IL-10 receptor α was applied to the cultured hippocampal neurons of DIV 3, and then RT-PCR was performed at DIV 7. The expression of IL-10 receptor α was decreased significantly by the application of shRNA. Quantification (shRNA/vector only): 15 A.U. shRNA, 0.79; 20 A.U. shRNA, 0.49; 25 A.U. shRNA, 0.20 (A.U., arbitrary unit). (B) Attenuated synaptic formation by application of shRNA of IL-10 receptor α. The induction of synaptic formation by microglia or recombinant IL-10 was dramatically attenuated when shRNA of IL-10 receptor α was applied to hippocampal neurons. Means±SEM. *n*=29 dendrites for vector only (shVec), 28 for shRNA of IL-10 receptor α #1 (shRNA#1), 26 for shRNA of IL-10 receptor α #2 (shRNA#2), 29 for the application of microglia only, 30 for microglia with shRNA#1, 26 for microglia with shRNA#2, 29 for the application of recombinant IL-10 (5 ng/ml) only, 30 for IL-10 with shRNA#1, 27 for IL-10 with shRNA#2. ***p<0.001 by the Newman-Keuls multiple comparison test after application of one-way ANOVA, *F*=23.04, *p*<0.0001. Scale bar, 10 μm.

A specific shRNA was applied to attenuate the expression of IL-10 receptors on the hippocampal neurons, and the effects of knockdown on synaptic formation were examined (Figure **4**). First, the efficiency of shRNA was evaluated by applying the lentivirus containing the shRNA of IL-10 receptor α to hippocampal neurons (Figure **4A**). The IL-10 receptor α mRNA was decreased considerably with application of the shRNA. Then two types of shRNA specific to rat IL-10 receptor α were applied to the hippocampal neurons of DIV 7, and the density of the dendrites was analyzed at DIV 15 (Figure **4B**). The induction of synaptic formation via microglia and recombinant IL-10 disappeared entirely after application of shRNA. These results confirmed again the expression of IL-10 receptors on hippocampal neurons, and the induction of synaptic formation by the IL-10 released from the microglia. Thus the developing microglia appear to regulate neuronal development through the release of IL-10 bound to IL-10 receptors that are expressed in hippocampal neurons.

### IL-1β antagonized IL-10 in neuronal synapse formation

**Figure 5:**
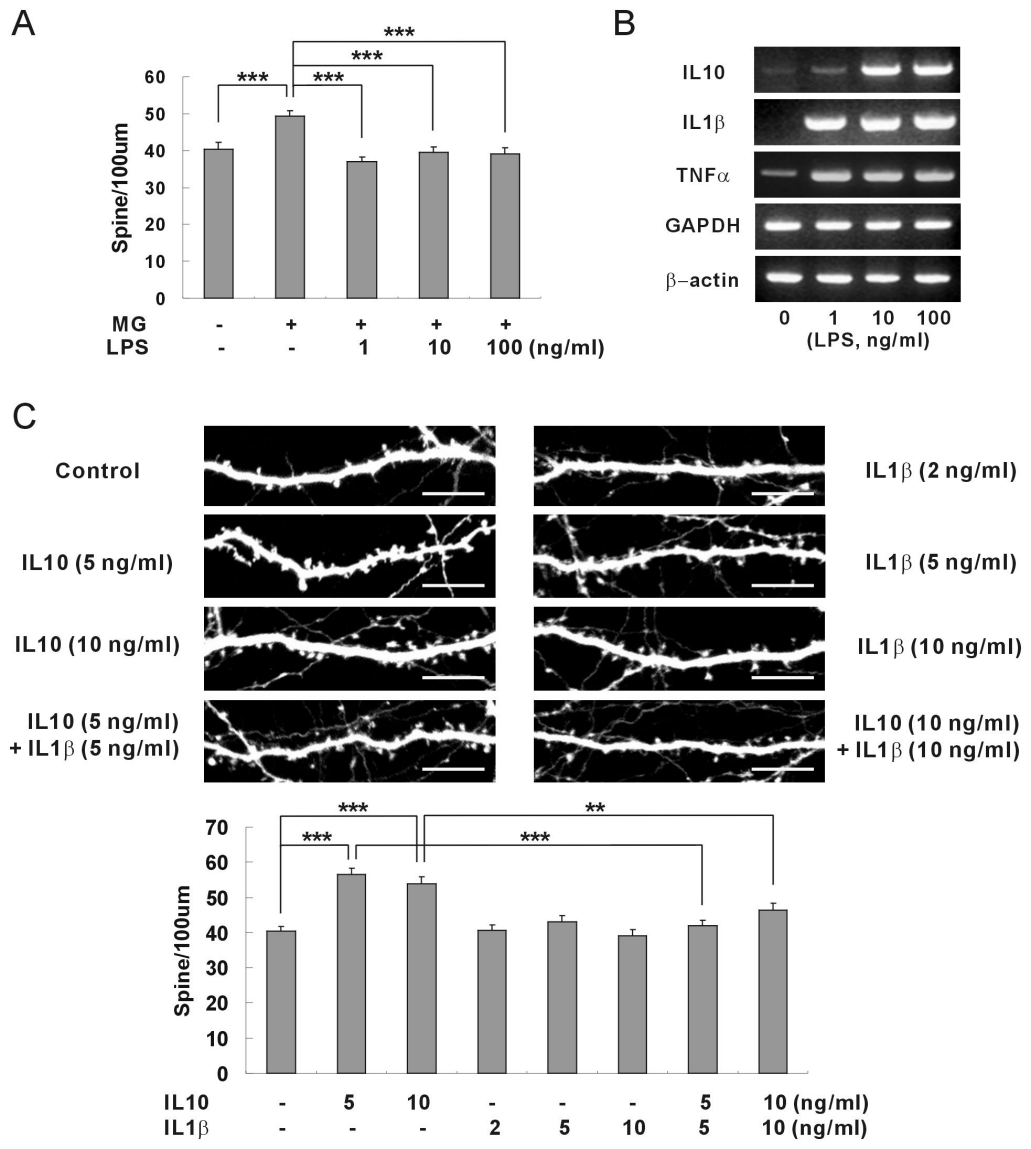
IL-1β antagonized IL-10 in neuronal synapse formation. (A) The induction of synaptic formation by microglia was attenuated when microglia were pretreated with lipopolysaccharide (LPS). Means±SEM. *n*=29 dendrites for no microglia, 29 for non-treated microglia, 30 for microglia pretreated with 1 ng/ml LPS, 30 for microglia pretreated with 10 ng/ml LPS, and 29 for microglia pretreated with 100 ng/ml LPS. ***p<0.001, by the Newman-Keuls multiple comparison test after application of one-way ANOVA, F=8.785, *p*<0.0001. (B) Increased expressions of cytokine mRNAs after LPS treatment. An RT-PCR was performed after the microglia were treated with LPS at indicated concentrations. The mRNAs of IL-1β and TNFα were increased significantly by 1 ng/ml LPS, but the expression of IL-10 mRNA was not convincingly increased by 1 ng/ml LPS. Quantification (LPS treated/not treated): IL-10 by 1 ng/ml LPS, 1.57; IL-10 by 10 ng/ml LPS, 4.13; IL-10 by 100 ng/ml LPS, 4.06; IL-1β by 1 ng/ml LPS, 19.2; IL-1β by 10 ng/ml LPS, 28.0; IL-1β by 100 ng/ml LPS, 25.8; TNFα by 1 ng/ml LPS, 4.26; TNFα by 10 ng/ml LPS, 5.07; TNFα by 100 ng/ml LPS, 4.48. (C) The induction of synaptic formation by IL-10 was attenuated when IL-1β was applied together with IL-10. Recombinant proteins of IL-10 or IL-1β were applied to the cultured hippocampal neurons of DIV 8 and the density of dendritic spines was analyzed after one week. IL-1β antagonized the induction of synaptic formation by IL-10. Means±SEM. *n*=30 dendrites for control (no IL-10 no IL-1β), 28 for 5 ng/ml IL-10, 27 for 10 ng/ml IL-10, 26 for 2 ng/ml IL-1β, 28 for 5 ng/ml IL-1β, 29 for 10 ng/ml IL-1β, 29 for 5 ng/ml IL-10 plus 5 ng/ml IL-1β, 30 for 10 ng/ml IL-10 plus 10 ng/ml IL-1β. **p<0.01 and ***p<0.001 by the Newman-Keuls multiple comparison test after application of one-way ANOVA, *F*=14.67, *p*<0.0001. Scale bar, 10 μm.

It is known that the release of cytokines from microglia can be enhanced in response to treatment with bacterial endotoxin LPS [5]. The effect of LPS treatment on neuronal synapse formation was examined to determine whether microglia could regulate synaptic formation under pathological conditions (Figure **5A**). When microglia were treated with LPS before application to hippocampal neurons, the induction of synaptic formation by microglia was attenuated. Treatment with 1 ng/ml LPS was sufficient to inhibit microglia from inducing synaptic formation. The RT-PCR assay showed that the expressions of IL-1β and TNFα on microglia were significantly increased by 1 ng/ml LPS, but on the other hand, IL-10 was not convincingly increased (Figure **5B**). Although IL-1β and TNFα did not have any effect on neuronal synapse formation (Figure **S2A**, **S2B**), they could interfere with IL-10 and inhibit the induction of synaptic formation by microglia. Thus recombinant IL-1β or TNFα was applied to hippocampal neurons, together with recombinant IL-10, in order to investigate the crosstalk between cytokines. The induction of synaptic formation by IL-10 disappeared when IL-1β was applied to hippocampal neurons (Figure **5C**). On the other hand, TNFα did not antagonize the effects of IL-10 on synaptic formation (Figure **S3**). These data suggest that the expression of IL-1β increased by LPS treatment attenuated the induction of synaptic formation by IL-10.

## Discussion

From the present study, the developing microglia were newly found to induce synapse formation of hippocampal neurons by releasing the anti-inflammatory cytokine IL-10. IL-10 appears to interact with IL-10 receptors expressed on hippocampal neurons of early developmental stage. Without external stimuli, microglia released an appreciable amount of IL-10 for the induction of synaptic formation. Pro-inflammatory cytokines IL-1β antagonized the functions of IL-10 in the pathological conditions. When the IL-10 receptors were knocked down in the hippocampal neurons, the induction of synaptic formation was significantly attenuated. In conclusion, microglia could regulate the synaptic formation by releasing IL-10 that interacts with IL-10 receptor expressed on the developing hippocampal neurons. 

Previously, cytokines such as TNFα, IL-1β, and IL-10 have been reported to regulate synaptic efficacy and synaptic plasticity, but the regulation mechanisms of cytokines were not well understood. Our results show that IL-10 induces synaptic formation of hippocampal neurons, and IL-1β antagonizes the effects of IL-10 when endogenously released from microglia or applied as recombinant proteins. It was reported that IL-1β receptors were also expressed on early postnatal neurons [31,34]. The developing microglia appear to regulate neuronal functions including synaptic formation and synaptic plasticity by releasing cytokines such as IL-10 and IL-1β of which receptors are expressed on early postnatal neurons.

Microglia have been reported to engulf synaptic inputs and to play a role in activity-dependent synaptic pruning during postnatal brain development [12,13,14]. These results which appear contradictory to ours, suggest that microglia function as a double-edged sword for synaptic formation: microglia could remodel developing synapses both through direct contact and by indirect processes. Microglia appear to engulf synaptic materials for synaptic pruning and concurrently to release cytokines for induction of synaptic formation. When microglia were applied “directly” to developing hippocampal neurons (DIV 8), the density of dendritic spines was “not” increased after one week (Figure **S4**). This result appears to make sense because the induction of synaptic formation by IL-10 released from microglia could be countervailed by synaptic pruning derived from direct contact with microglia. In this context, it appears to be answerable for a recent report that synaptic formation was inhibited by “direct” application of microglia to the matured neurons [35]. Because IL-10 receptors are not expressed in the matured neurons, synaptic pruning could prevail and synaptic formation would be attenuated. When recombinant IL-10 was applied to the matured hippocampal neurons of DIV 14, synaptic formation was “not” induced after one week (DIV 21) (Figure **S5**). According to our results, the expression of IL-10 receptor α was significantly decreased in hippocampal neurons of DIV 15 (Figure **3A-3C**). Therefore, IL-10 could regulate neuronal synapse formation in the early brain development. 

Recently, IL-10 has attracted significant attention for potential therapeutic application because of its anti-inflammatory and immunosuppressive effects [27,36,37,38]. IL-10 was suggested to have important roles for neuronal protection in glutamate-mediated neuronal cell death, focal brain ischemia, and spinal cord injuries. After central nervous system injuries, IL-10 was shown to improve neurological outcomes by reducing the number of apoptotic cells [36]. In transgenic mice expressing murine IL-10, the release of pro-inflammatory cytokines including TNFα, interferon-γ, and IL-1β were significantly reduced and the infarct size was reduced compared with wild type mice for middle cerebral artery occlusion [37]. The injection of herpes simplex virus-based vectors to express IL-10 in the spinal cord, increased the neuronal survival in the anterior quadrant of the spinal cord, and improved motor function after injury [33]. IL-10 appears to protect brains and to assist with neuronal functions under normal physiological conditions as well as in emergency situations.

Neurons were considered to be immune-privileged in a healthy brain; however, it has been known that the major factors of the immune system have significant roles in neuronal functions. Among them, MHC class I was suggested to regulate synaptic functions in the developing visual system, and to play major roles especially in postsynaptic activity [39,40,41]. In addition, C1q, the initiating protein of the classical complement pathway, was also shown to localize to immature synapses with C3 and necessary for the developmental pruning of retinogeniculate synapses [42]. IL-6, a pro-inflammatory cytokine, was reported to inhibit the induction LTP in hippocampal slices [43]. These results suggest that many factors of immune systems have major roles in the maintenance of synaptic plasticity as well as in the regulation of synaptic formation [44,45,46]. Therefore, it would be very interesting to discover new roles of immune components including various cytokines and chemokines in neuronal functions and brain development.

## Materials and Methods

### Ethics statement

This study was performed in accordance with the regulations outlined by the Korean law. The animal experiment protocols were approved by the Animal Use and Care Committee of Korea Research Institute of Bioscience and Biotechnology (Permit Number: KRIBB-AEC-11022). Animals were sacrificed using CO_2_ gas, and all efforts were made to minimize suffering.

### Cytokines and antibodies

Recombinant cytokines were purchased from R&D systems (Minneapolis, MN, USA): rat IL-10 (547-RL), rat IL-1β (501-RL), rat TNFα (510-RT), rat IL-6 (506-RL), and rat IL-4 (504-RL). The following antibodies were used: anti-mouse IL-10 receptor α (AF-474-NA), anti-rat TNFα (MAB510) obtained from R&D systems (Minneapolis, MN, USA); anti-CD11b/c (ab1211), anti-GFAP (ab16997) from Abcam (Cambridge, UK); anti-vGLUT (135 303), anti-vGAT (131 002) from SYSY (Gottingen, Germany); anti-Iba-1 from Wako Pure Chemical Industries (Osaka, Japan); anti-MAP2 (M1406) from Sigma-Aldrich (Saint Louis, MO, USA); anti-β-actin (4967) from Cell Signaling (Danvers, MA, USA); anti-IL-10 receptor α (sc-985) from Santa Cruz Biotechnology (Dallas, TX, USA) [47,48]. The neutralizing antibody of IL-10 receptor α (R&D systems, AF-474-NA) or anti-rat TNFα antibody (R&D systems, MAB510), was applied to the culture media at a concentration of 2 μg/ml.

### Primer for RT-PCR

In these experiments, 1 μg of total RNA was reverse transcribed in a 20 μl reaction volume. PCRs were carried out using 1 μl of the completed RT as template with the corresponding primers: rat IL-10 mRNA (NM_012854), 5’- TGCCTTCAGTCAAGTGAAGAC-3’ (forward primer) and 5’-AAACTCATTCATGGCCTTGTA-3’ (reverse primer); rat IL-1β (NM_031512), 5’-CACCTTCTTTTCCTTCATCTTTG-3’ (forward primer) and 5’-GTCGTTGCTTGTCTCTCCTTGTA-3’ (reverse primer); rat TNFα (NM_012675), 5’-CCCAGACCCTCACACTCAGAT-3’ (forward primer) and 5’-TTGTCCCTTGAAGAGAACCTG-3’ (reverse primer); rat IL-10 receptor α (NM_057193), 5’-CTCGCTTCACAGTGGATGAA-3’ (forward primer) and 5’-TAAATACGGTGGTGCGTGAA-3’ (reverse primer); rat IL-10 receptor β (NM_00110711), 5’-TCAGCATGGTGTGGTTCATT-3’ (forward primer) and 5’-TCTTCCGTGATGATGCTCAG-3’ (reverse primer); rat Iba-1 (NM_017196), 5’-CCATGAAGCCTGAGGAAATTTCA-3’ (forward primer) and 5’-TTATATCCACCTCCAATTAGGGCA-3’(reverse primer); rat GFAP (NM_017009), 5’-GAAACCAACCTGAGGCTGGAG-3’ (forward primer) and 5’-GGCGATAGTCATTAGCCTCG-3’ (reverse primer); rat GAPDH (NM_017008), 5’-CCCCCAATGTATCCGTTGTG-3’ (forward primer) and 5’-TAGCCCAGGATGCCCTTTAGT-3’ (reverse primer); rat β-actin (NM_031144), 5’-AGAAGAGCTATGAGCTGCCTGACG-3’ (forward primer) and 5’-TACTTGCGCTCAGGAGGAGCAATG-3’ (reverse primer).

### Production of shRNA of IL-10 receptor α

For knockdown of rat IL-10 receptor α (NM_057193), nt 738 - 756 (CGTGGAATCCCGAATTAAC, shRNA of IL-10 receptor α #1) or nt 1429 - 1447 (TACCAGAAGCAGACCAGAT, shRNA of IL-10 receptor α #2) was subcloned into pSuper.gfp/neo plasmid vector (OligoEngine, Seattle, WA, USA). pSuper.gfp/neo containing shRNA was transfected and expressed in rat cultured hippocampal neurons using the calcium phosphate method. The same sequence for shRNA was used for the production of lentivirus. 

### Preparation of mixed glial cell culture and harvest of floating microglia

Rats of postnatal 1 day were decapitated and the skulls were washed twice using pre-warmed L-15 media (Sigma, Saint Louis, USA) with penicillin/streptomycin. The brains were taken out of skulls, washed twice, and stripped of their meninges. Cortex were isolated without hippocampus, transferred to conical tube, and minced with Pasteur pipette in L-15 media. Minced cortex was centrifuged at 1200 rpm for 3 sec and the supernatant containing cells were transferred to new conical tube. Then the pellet was added with new L-15 media, re-suspended, and centrifuged again. The resulted supernatant was transferred to new conical tube, and this process was performed once more. The pooled supernatant containing cells was centrifuged at 1400 rpm 5 min and the resulted pellet was added with the glial media (DMEM media with 10% FBS, 1 mM L-glutamine, 1 mM sodium pyruvate, penicillin/streptomycin). The glial cells were re-suspended and transferred to cell culture dish pre-coated with poly-D-lysine. After 1 day, half of media was changed with new glial media and then twice in a week. The floating microglia cells were harvested at 7~14 days after plating and centrifuged at 1400 rpm 5 min. The pellet was re-suspended with the glial media and transferred to porous cell culture insert (Millicell-PCF, PIHP01250, Millipore, USA). The prepared microglia were stabilized on cell culture insert overnight and then applied to cultured hippocampal neurons next day.

### Preparation of cultured hippocampal neurons, transfection of neurons, and immunohistofluorescence

Primary hippocampal neurons were prepared from embryonic day 18 (E18) rats, grown on glass coverslip pre-coated with poly-D-lysine in serum-free neurobasal media (Invitrogen, USA) with glutamine and B-27 serum-free supplement (Invitrogen, USA), and transfected using the calcium phosphate method at days in vitro (DIV) 7, as described previously [49]. Microglia plated on porous cell culture insert or IL-10 were applied to hippocampal neurons of DIV 8. After one week, at DIV 15, hippocampal neurons were fixed in 4% (v/v) formaldehyde/4% (w/v) sucrose, permeabilized with 0.2% (v/v) Triton X-100 in phosphate-buffered saline, incubated with primary antibodies (anti-EGFP, anti-vGLUT, anti-vGAT, anti-IL-10 receptor α, anti-MAP2, 1~5 μg/ml) overnight at 4 °C, and finally incubated with Cy3-, or FITC-conjugated secondary antibodies (1:1000, or 1:250 dilution) (Jackson ImmunoResearch Laboratories, West Glove, PA, USA) for 2 h at room temperature. Microglia was fixed, permeabilized, incubated with primary antibodies (anti-CD11b/c, anti-GFAP) overnight at 4 °C, and finally incubated with Cy3-, or FITC-conjugated secondary antibodies for 2 h at room temperature.

### Image acquisition and quantification

Images captured by confocal microscopy (LSM 510 Meta, Zeiss, Gottingen, Germany) using a 63x objective were analyzed blindly using MetaMorph software (Universal Imaging) [50]. The density of dendritic spines (0.4-2.5 μm) and synaptic protein clusters were measured from 30-40 dendrites of eight to ten neurons; the total dendritic length of ~50 μm was measured from the first dendritic branching points. Means from multiple individual dendrites were averaged to obtain a population mean and SEM. All experiments were repeated more than three times with similar results.

### Sample preparation for western blot analysis

Primary hippocampal neurons grown on cell culture dishes were lysed with ice-cold 1% (v/v) Triton X-100 in Dulbecco’s phosphate-buffered saline (pH 7.4) (Invitrogen, USA) containing inhibitors of proteases and phosphatases. After incubation on ice for 30 min, the neuron lysates were centrifuged at 12,000 rpm for 30 min at 4 °C. The cleared extracts were mixed with SDS-PAGE sampling buffer, boiled for 8 min, resolved on SDS-PAGE, and blotted to nitrocellulose membrane (Amersham, UK). After incubated using primary antibodies (anti-IL-10 receptor α, anti-Iba-1, anti-GFAP, anti-β-actin, anti-PSD-95, anti-α-tubulin, 0.2~0.5 μg/ml) and then HRP-labeled secondary antibodies, the protein bands blotted on nitrocellulose membranes were detected with ECL (Pierce chemical co, USA).

Mixed glial cells grown on cell culture dishes were lysed with ice-cold 1% (v/v) Triton X-100 in Dulbecco’s phosphate-buffered saline containing inhibitors of proteases and phosphatases. The mixed glial lysates were cleared, and mixed with SDS-PAGE sampling buffer. Floating microglia were harvested, centrifuged, and lyzed with ice-cold 1% (v/v) Triton X-100 in Dulbecco’s phosphate-buffered saline containing inhibitors of proteases and phosphatases. 

For the study of developing rat brain, brains were removed from embryonic day 18 (E18), postnatal day 1, 3, 7, 14 (P1, P3, P7, P14 respectively), and postnatal 3, 6 weeks (P3W and P6W respectively) rats. Brains were lysed in ice-cold buffered sucrose (0.32 M sucrose, 4 mM HEPES, 1 mM MgCl_2_, 0.5 mM CaCl_2_, pH 7.3) with protease inhibitors using a tissue homogenizer, mixed with SDS-PAGE sampling buffer, resolved on SDS-PAGE, and blotted to nitrocellulose membrane. 

### Measurement of IL-10 released from microglia

The microglia prepared from the mixed glial culture were plated on a porous cell culture insert, and then incubated in a neuronal culture media. After 3~7 days, the amount of IL-10 released from the microglia was analyzed. The amount of IL-10 released from the mouse microglia was measured using an ELISA kit (BD OptEIA Set Mouse IL-10, BD Biosciences, San Diego, CA, USA). The ELISA assay was carried out according to manufacturer instructions. Briefly, standard or culture media containing IL-10 were added to each well of the ELISA plate, pre-bound with anti-IL-10 antibody, incubated for 2 hr at room temperature, and washed five times. Then detection antibody and HRP were incubated successively. Finally, TMB substrate solution was added, incubated for 30 min in the dark, and the absorbance was measured at 450 nm with λ correction 570 nm.

### Treatment of microglia with LPS

Microglia on cell culture inserts were treated with LPS for 4 h and then residual LPS was washed out before microglia was applied to the cultured hippocampal neurons of DIV 8. After one week, at DIV 15, the synaptic formation of hippocampal neurons was analyzed.

### Statistics

Results are expressed as Means±SEM and one-way ANOVA followed by application of the Newman-Keuls multiple comparison test was used to assess statistical significance. A comparison was considered to be significant if *P*<0.05.

## Supporting Information

Figure S1 **Neuronal synapse formation not induced through purinergic receptor.** Reactive blue (RB), the antagonist of P2Y purinergic receptor, was added to the co-culture system of hippocampal neurons and microglia. Synaptic formation was not reduced by treatment with RB. Means±SEM. *n*=40 dendrites for no microglia, 40 for 1.0 × 10^5^ microglia, 40 for 1.0 × 10^5^ microglia plus 5 ng/ml RB, 40 for 1.0 × 10^5^ microglia plus 50 ng/ml RB. ***p<0.001, by the Newman-Keuls multiple comparison test after application of one-way ANOVA, *F*=29.16, *p*<0.0001. Scale bar, 10 μm.
(TIF)

Figure S2 **Neuronal synapse formation not induced by various cytokines.** (A) The density of dendritic spines was not increased by application of recombinant IL-1β. Recombinant proteins of IL-1β were applied to the neuronal culture media at DIV 8 and after one week the density of dendritic spines was analyzed. Means±SEM. *n*=29 dendrites for control (no IL-1β), 30 for 2 ng/ml IL-1β, 30 for 5 ng/ml IL-1β, 29 for 10 ng/ml IL-1β, 29 for 20 ng/ml IL-1β. These differences are considered to be not statistically significant by the Newman-Keuls multiple comparison test after application of one-way ANOVA, *F*=1.100, *p*=0.3591.
(B) The application of the recombinant TNFα did not induce synaptic formation significantly at any concentration. Means±SEM. *n*=28 dendrites for control (no TNFα, 28 for 0.03 ng/ml TNFα, 28 for 0.3 ng/ml TNFα, 28 for 3 ng/ml TNFα, 30 for 10 ng/ml TNFα, 30 for 100 ng/ml TNFα These differences are considered to be not statistically significant by the Newman-Keuls multiple comparison test after application of one-way ANOVA, *F*=1.736, *p*=0.1291.
(C) The application of the recombinant IL-6 did not induce synaptic formation significantly at any concentration. Means±SEM. *n*=29 dendrites for control (no IL-6), 31 for 0.5 ng/ml IL-6, 30 for 5 ng/ml IL-6, 29 for 20 ng/ml IL-6, 29 for 400 ng/ml IL-6. These differences are considered to be not statistically significant by the Newman-Keuls multiple comparison test after application of one-way ANOVA, *F*=2.227, *p*=0.0690.
(D) The application of the recombinant IL-4 did not induce synaptic formation significantly at any concentration. Means±SEM. *n*=27 dendrites for control (no IL-4), 28 for 2 ng/ml IL-4, 27 for 5 ng/ml IL-4, 29 for 10 ng/ml IL-4. These differences are considered to be not statistically significant by the Newman-Keuls multiple comparison test after application of one-way ANOVA, *F*=2.343, *p*=0.0772.
(TIF)

Figure S3 **TNFα did not antagonize the effects of IL-10.** When TNFα was applied to hippocampal neurons together with IL-10, the induction of synaptic formation by IL-10 was not attenuated. TNFα could not antagonize the effects of IL-10 in synaptic formation. Means±SEM. *n*=28 dendrites for control (no IL-10 no TNFα), 29 for 5 ng/ml IL-10, 29 for 5 ng/ml TNFα, 30 for 5 ng/ml IL-10 plus 5 ng/ml TNFα. *p<0.05 and **p<0.01, by the Newman-Keuls multiple comparison test after application of one-way ANOVA, *F*=23.32, *p*<0.0001.
(TIF)

Figure S4 **Direct application of microglia did not induce synaptic formation.** When developing microglia were applied directly to the hippocampal neurons of DIV 8 and synaptic formation was analyzed after one week, the density of dendritic spine was not increased. Means±SEM. *n*=29 dendrites for control (no microglia), 28 for 1.0 × 10^5^ microglia plated on cell culture insert, 28 for 0.1 × 10^5^ microglia applied directly, 29 for 0.5 × 10^5^ microglia applied directly, 29 for 1.0 × 10^5^ microglia applied directly. ***p<0.001, by the Newman-Keuls multiple comparison test after application of one-way ANOVA, *F*=11.73, *p*<0.0001.
(TIF)

Figure S5 **IL-10 did not induce synaptic formation in the matured neurons.** When recombinant IL-10 (5 ng/ml) was applied to hippocampal neurons of DIV 14 and synaptic formation was analyzed after one week (DIV 21), the density of dendritic spine was not increased significantly. Means±SEM. *n*=28 dendrites for control (no IL-10), 27 for 5 ng/ml IL-10. These differences are considered to be not statistically significant by the Newman-Keuls multiple comparison test after application of one-way ANOVA, *F*=2.022, *p*=0.2281.
(TIF)
