# Supplementary figures and images for: Neuronal Synapse Formation Induced by Microglia and Interleukin 10
Source: PLoS One. 2013 Nov 22;8(11):e81218. doi: 10.1371/journal.pone.0081218 (PMC3838367; doi:10.1371/journal.pone.0081218)

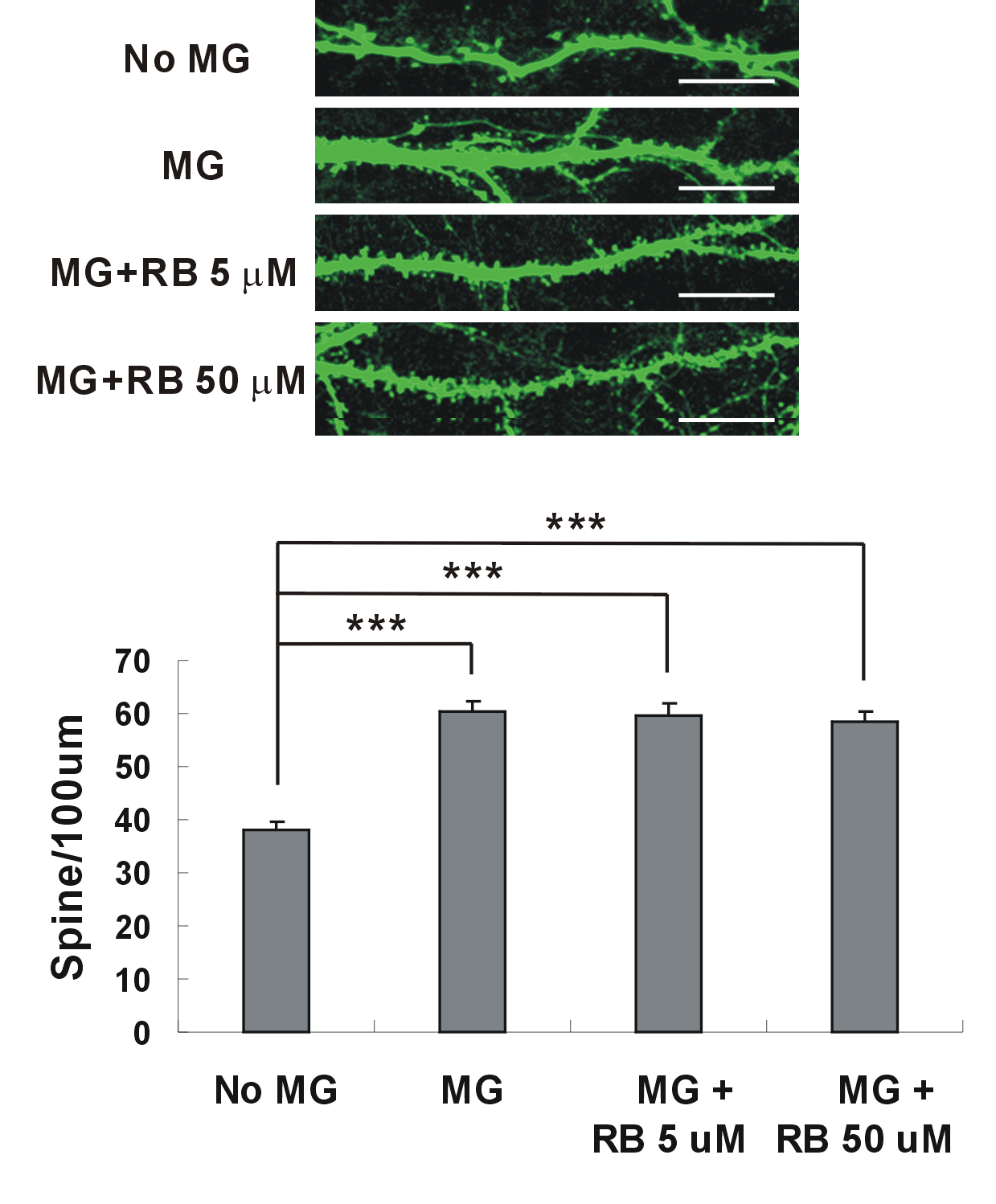

Supplement: Figure S1 — Neuronal synapse formation not induced through purinergic receptor. Reactive blue (RB), the antagonist of P2Y purinergic receptor, was added to the co-culture system of hippocampal neurons and microglia. Synaptic formation was not reduced by treatment with RB. Means±SEM. n=40 dendrites for no microglia, 40 for 1.0 × 10^5^ microglia, 40 for 1.0 × 10^5^ microglia plus 5 ng/ml RB, 40 for 1.0 × 10^5^ microglia plus 50 ng/ml RB. ***p<0.001, by the Newman-Keuls multiple comparison test after application of one-way ANOVA, F=29.16, p<0.0001. Scale bar, 10 μm. (TIF) [file pone.0081218.s001.tif]

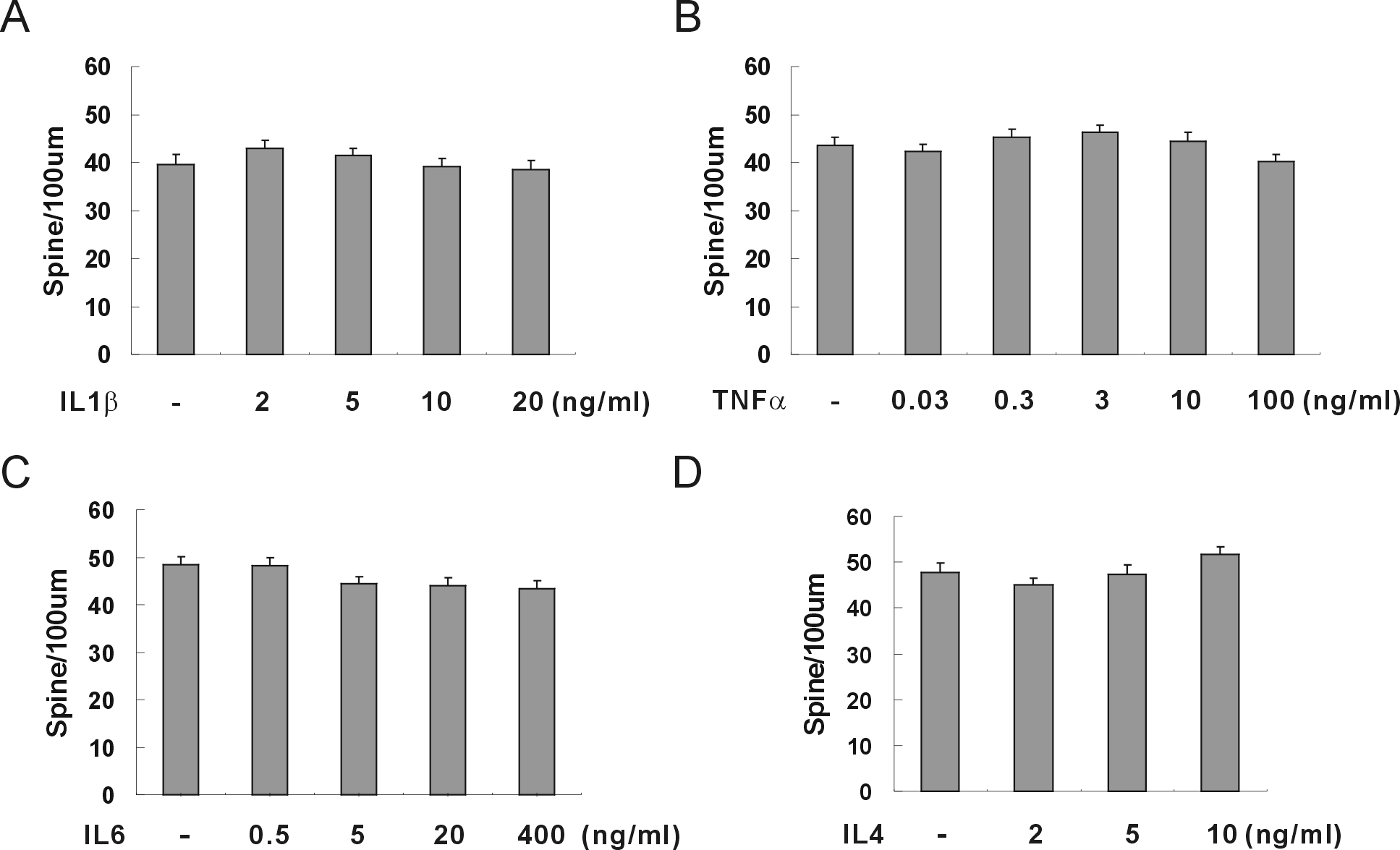

Supplement: Figure S2 — Neuronal synapse formation not induced by various cytokines. (A) The density of dendritic spines was not increased by application of recombinant IL-1β. Recombinant proteins of IL-1β were applied to the neuronal culture media at DIV 8 and after one week the density of dendritic spines was analyzed. Means±SEM. n=29 dendrites for control (no IL-1β), 30 for 2 ng/ml IL-1β, 30 for 5 ng/ml IL-1β, 29 for 10 ng/ml IL-1β, 29 for 20 ng/ml IL-1β. These differences are considered to be not statistically significant by the Newman-Keuls multiple comparison test after application of one-way ANOVA, F=1.100, p=0.3591. (B) The application of the recombinant TNFα did not induce synaptic formation significantly at any concentration. Means±SEM. n=28 dendrites for control (no TNFα, 28 for 0.03 ng/ml TNFα, 28 for 0.3 ng/ml TNFα, 28 for 3 ng/ml TNFα, 30 for 10 ng/ml TNFα, 30 for 100 ng/ml TNFα These differences are considered to be not statistically significant by the Newman-Keuls multiple comparison test after application of one-way ANOVA, F=1.736, p=0.1291. (C) The application of the recombinant IL-6 did not induce synaptic formation significantly at any concentration. Means±SEM. n=29 dendrites for control (no IL-6), 31 for 0.5 ng/ml IL-6, 30 for 5 ng/ml IL-6, 29 for 20 ng/ml IL-6, 29 for 400 ng/ml IL-6. These differences are considered to be not statistically significant by the Newman-Keuls multiple comparison test after application of one-way ANOVA, F=2.227, p=0.0690. (D) The application of the recombinant IL-4 did not induce synaptic formation significantly at any concentration. Means±SEM. n=27 dendrites for control (no IL-4), 28 for 2 ng/ml IL-4, 27 for 5 ng/ml IL-4, 29 for 10 ng/ml IL-4. These differences are considered to be not statistically significant by the Newman-Keuls multiple comparison test after application of one-way ANOVA, F=2.343, p=0.0772. (TIF) [file pone.0081218.s002.tif]

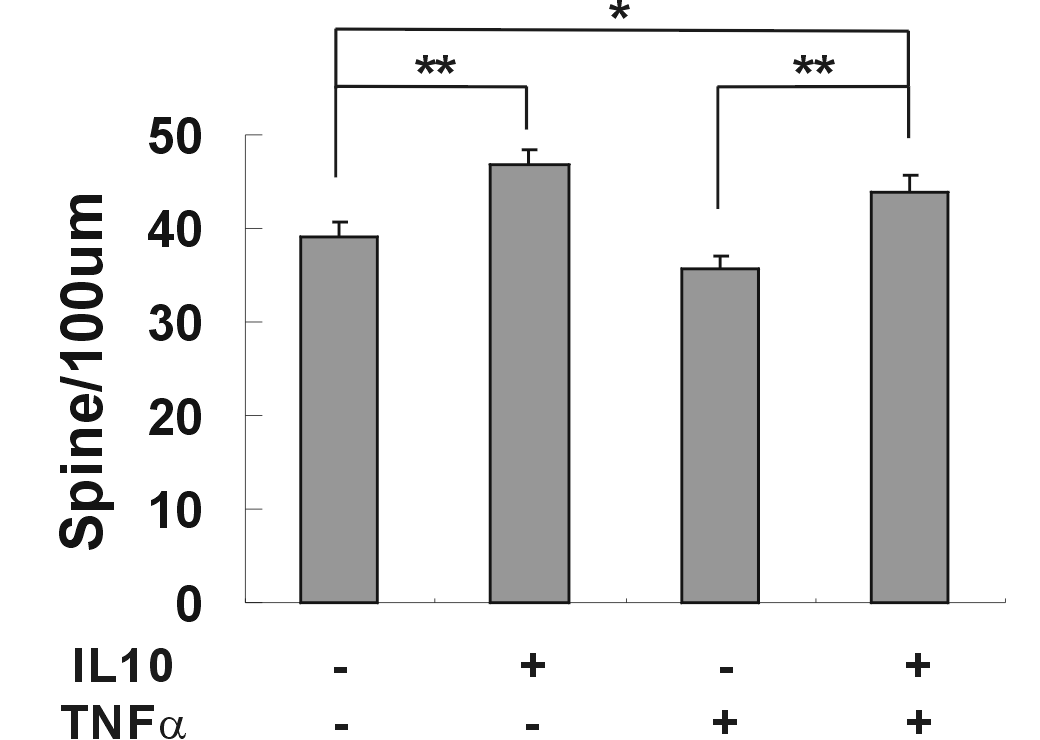

Supplement: Figure S3 — TNFα did not antagonize the effects of IL-10. When TNFα was applied to hippocampal neurons together with IL-10, the induction of synaptic formation by IL-10 was not attenuated. TNFα could not antagonize the effects of IL-10 in synaptic formation. Means±SEM. n=28 dendrites for control (no IL-10 no TNFα), 29 for 5 ng/ml IL-10, 29 for 5 ng/ml TNFα, 30 for 5 ng/ml IL-10 plus 5 ng/ml TNFα. *p<0.05 and **p<0.01, by the Newman-Keuls multiple comparison test after application of one-way ANOVA, F=23.32, p<0.0001. (TIF) [file pone.0081218.s003.tif]

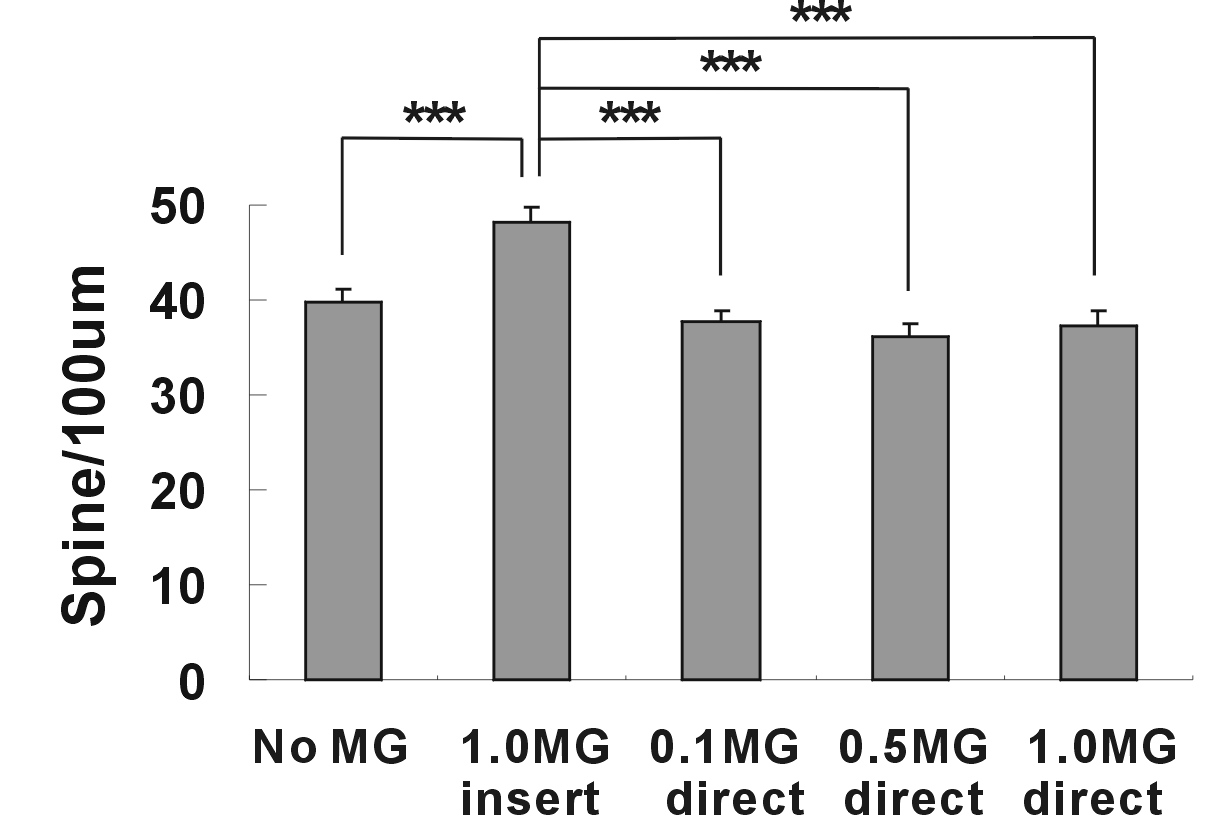

Supplement: Figure S4 — Direct application of microglia did not induce synaptic formation. When developing microglia were applied directly to the hippocampal neurons of DIV 8 and synaptic formation was analyzed after one week, the density of dendritic spine was not increased. Means±SEM. n=29 dendrites for control (no microglia), 28 for 1.0 × 10^5^ microglia plated on cell culture insert, 28 for 0.1 × 10^5^ microglia applied directly, 29 for 0.5 × 10^5^ microglia applied directly, 29 for 1.0 × 10^5^ microglia applied directly. ***p<0.001, by the Newman-Keuls multiple comparison test after application of one-way ANOVA, F=11.73, p<0.0001. (TIF) [file pone.0081218.s004.tif]

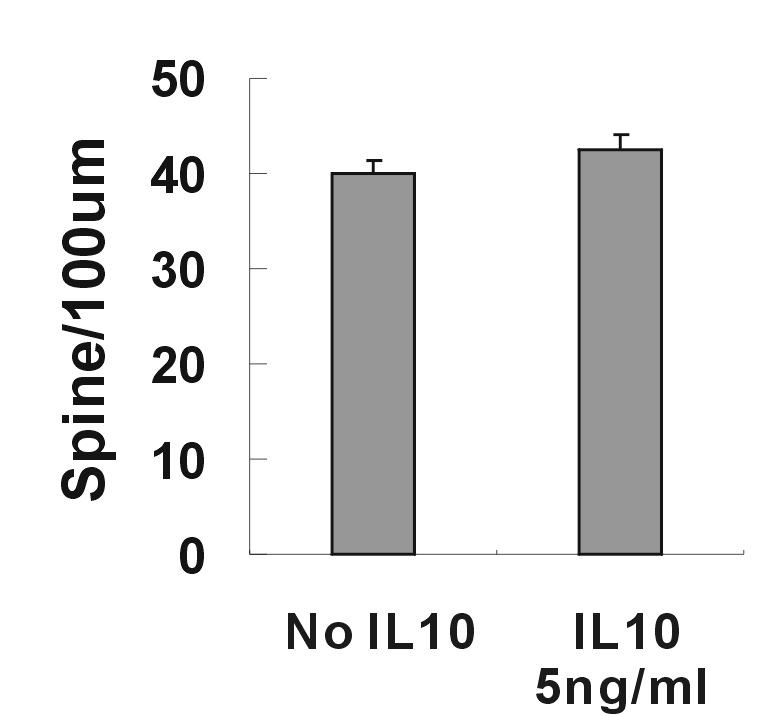

Supplement: Figure S5 — IL-10 did not induce synaptic formation in the matured neurons. When recombinant IL-10 (5 ng/ml) was applied to hippocampal neurons of DIV 14 and synaptic formation was analyzed after one week (DIV 21), the density of dendritic spine was not increased significantly. Means±SEM. n=28 dendrites for control (no IL-10), 27 for 5 ng/ml IL-10. These differences are considered to be not statistically significant by the Newman-Keuls multiple comparison test after application of one-way ANOVA, F=2.022, p=0.2281. (TIF) [file pone.0081218.s005.tif]
